# The influence of informal caregivers on the place of death: A study within a home palliative care team

**DOI:** 10.1177/26323524251336764

**Published:** 2025-06-06

**Authors:** Cristina Pereira, Hugo Ribeiro, Júlia Magalhães, João Rocha-Neves, Marília Dourado, José Paulo Andrade

**Affiliations:** 1 Community Palliative Care Support Team Gaia, Vila Nova de Gaia, Portugal; 2 Faculty of Medicine of University of Porto, Portugal; 3 Faculty of Medicine of University of Coimbra, Portugal; 4 Center for Innovative Biomedicine and Biotechnology, Coimbra, Portugal; 5 RISE@Health, Porto, Portugal

**Keywords:** palliative care, place of death, quality of care, caregiver perspectives

## Abstract

**Background:**

The increase in the prevalence of chronic, complex, or life-limiting diseases is intrinsically associated with population aging. Therefore, it is necessary to reflect on health and social care, and community palliative care can play a fundamental role in responding to this phenomenon.

**Objective:**

This research aimed to understand the factors that affect the care place at the end of life and death, identifying the informal caregivers’ profile, the challenges of the all-care process, and the impact of a Community Palliative Care Team (CPCT).

**Design:**

An observational retrospective cohort study was carried out.

**Methods:**

The population of this study was caregivers of patients monitored by a Community Palliative Care Support Team between 2020 and 2022. In the sample, 78 caregivers were included according to inclusion and exclusion criteria, of whom 44 caregivers answered, representing a 55.70% response rate.

**Results:**

Although it is not possible to generalize as we do not have statistically significant correlations, this study concludes that the informal caregivers included in the sample have a profile similar to the national profile in Portugal. They reported high emotional exhaustion, but also physical and financial exhaustion, as the primary care challenges. In addition, the results show that managing the progression of the disease was also highlighted as a challenge by 63.6% of caregivers. The impact of the team monitoring was also evaluated as positive and having an effect in achieving the patient’s preference regarding the place of death.

**Conclusion:**

CPCT and informal caregivers are two factors intrinsically linked and influencing the place of death at home when there is congruence between the patients and their caregivers’ choices. Social resources and investment in a support network outside the hospital are essential to fulfilling the wishes of patients and families, allowing dignity and quality of life for both. From a medical point of view, these patients are so well or better treated in the community than in the hospital wards.

## Introduction

Population aging is a global phenomenon with significant social transformations and profound consequences for all sectors of society, particularly family structure, social protection, and health care.^1^

Community palliative care (CPC) could play a vital role, concomitantly with social protection measures. These measures may include financial support for dependencies, human technical support in hygiene and nutrition, or differentiated technical support, such as articulated beds or adaptations to the home according to individual dysfunction.^2^ A study carried out by the OECD that analyzed social measures in 25 countries, evaluating their impact on long-term care, concluded that most countries provide financial support and reinforce contributions for consumables and medicines to older, more dependent, and sicker patients. The calculation formulas for obtaining these benefits are different, although they concluded that they are insufficient for the poorest populations, and we end up having more people in a situation of poverty when they have a very sick family member at home that they want to take care of.^3^

It is known that in Europe, more than 50% of people prefer to have end-of-life care and die at home. However, only a third of all deaths occur at home. Several factors affect the place of death, making identifying and analyzing them in their diversity and correlation essential to understanding this phenomenon better.^4^

According to the World Health Organization, chronic diseases represent around 60% of total deaths worldwide and are responsible for 80% of the total years lived with disability.^5^ It is predictable that a long period of illness and functional limitations precede death. This fact has aroused growing concern regarding the place where end-of-life care is provided and the place of death, mainly due to the need to identify and define appropriate responses for this population.^5^

Only 14% of the world’s population has access to a specialized palliative care (PC). The proportion of patients with access to a specialized PC at home is still being determined.^6^

Previous studies identified the combination of factors such as patient preference,^7–9^ family member/caregiver preference,^7–11^ and marital status^12^ as consistent elements for death to occur at home.^8,9,11^ Other studies concluded that having home PC^7,11,13–16^ is decisive.

PC plays a critical role in addressing the needs of patients with chronic, complex, or life-limiting conditions, particularly in community settings. This study explores how CPC teams influence caregivers’ experiences and preferences regarding the place of death, emphasizing the interplay between caregiving dynamics and care preferences. The preferred place of death can result from a complex and dynamic process involving individual characteristics, disease characteristics, community characteristics, and government policies.^17,18^

This study aims to explore the perspectives of informal caregivers on the factors influencing the place of death for patients receiving PC at home. Specifically, it seeks to identify caregivers’ profiles, the challenges encountered throughout the care process, and the impact of a CPC team in facilitating end-of-life care. This team has several high-performance indicators, such as the quick start of follow-up after referral, which happens in less than 24 h, and the notable improvement within 2 weeks in obtaining psychological support, basic social support, and in controlling symptoms and therapeutic adaptation to patients’ current targets.^19^

It also had the following secondary objectives: (a) identify and evaluate the sociodemographic characteristics of the caregiver; (b) identify the association between kinship and motivation for care; (c) identify the needs and potential of caregivers who influenced the choice of place of death during the care journey.

## Methods

### Study design

Observational, analytical, retrospective cohort, empirical study using the quantitative method.

We have followed the Strengthening the Reporting of Observational Studies in Epidemiology (STROBE) guideline^20^ (Supplemental File with STROBE checklist).

### Selection of participants

Caregivers of patients monitored by a Portuguese Community Palliative Care Team (CPCT) between the years 2020 and 2022 inclusive.

Regarding the determination and definition of the sample, it was based on the following inclusion criteria: be the main caregiver for providing care to a patient accompanied by the CPCT for a minimum period of 1 month; be 18 years of age or older; know how to read and write; patients and caregivers wish for the death to occur at home (their own, family or friends).

An exclusion criterion was also considered: users institutionalized in a National Network of Integrated Continued Care and National Palliative Care Network units, hospitals, and Residential Structures for the Elderly.

Caregivers were contacted by telephone to be informed about the study and its objectives. Caregivers who expressed their intention to participate were asked for their preferred response: via email, at the caregiver’s home, in person, or another method proposed by the caregiver. The location/route of completion was the caregiver’s preference.

There is a potential impact of the COVID-19 pandemic on selection and results, as in the majority of responses, we see a preference for completing the questionnaire in person. Having not assessed the causes of refusal to participate in the study, we can assume that given the turbulent period, we were experiencing at the time, nonparticipation may have been related to restrictions related to the pandemic or fears about interacting with healthcare personnel.

### Data collection

The study took place between February and June 2023.

The researchers constructed a questionnaire to collect information, consisting of 38 questions (Annex). The questionnaire was initially reviewed and revised by three subject matter experts having extensive experience in consolidating and validating questionnaires. Then, it was administered to a pilot group of 20 participants (healthcare professionals) for testing, and then it was administered to another pilot group of 20 participants from a caregiver association. Based on the feedback received, it was subsequently restructured and applied to the final study population. The questions are closed, multiple choice, which allows the selection of one or more answers among the options presented, as well as adding a new answer that was not considered by the researchers and a 5-point Likert answer (1 = Bad and 5 = Excellent) to assess caregivers’ satisfaction with the care provided by the CPCT. The questionnaire, on paper, aimed to identify the profile of caregivers through their sociodemographic characterization, characterization of health care, time spent providing care, information regarding the patient’s preferred place of death, of the caregiver, and the actual place of death, identify informal and formal support that they received and/or that they identify as being necessary. Regarding the monitoring of CPCT, the aim is to identify potentialities and limitations inherent to its operation, as well as whether it was a condition for choosing and maintaining the place of death of the patient and/or caregiver.

For sociodemographic characterization, we analyzed gender, marital status, age, level of education, and professional status. Regarding the primary caregiver, we analyzed kinship with the patient, length of relationship with the patient, time as caregiver, and time spent on tasks. We analyzed the support that the patient/caregiver benefited from, categorizing it into informal and formal support, and the main challenges perceived as a caregiver. About the place of death, the patient’s preferred place of death, the caregiver’s preferred place of death, congruence between the patient’s preferred place of death and the caregiver’s preferred place of death, and congruence between the desired and actual place of death were analyzed. As a limitation, it is important to stress that the patient’s preferred place of death was reported by caregivers based on prior discussions or expressed wishes.

Regarding the characterization of health care, the limitations and potential of the CPCT were researched using the 5-point Likert scale. Through a dichotomous question (yes or no), the aim is to evaluate the influence of PC monitoring for death on the occurrence of death at home.

The European General Data Protection Regulation (GDPR) was respected,^21^ and the identifiable data was anonymized and protected by a password.

### Data analysis

Data were exported from the paper questionnaire to a Microsoft Excel^®^ database to interpret the results. Statistical analysis involved descriptive statistics measures (absolute and relative frequencies) and inferential statistics. The Spearman correlation coefficient, the Student’s *t* test for one sample, and the Fisher test were used. The significance level to reject the null hypothesis was set at α ⩽ 0.05. Statistical analysis was done using Statistical Package for the Social Sciences (SPSS) software version 28.0 for Windows (IBM^®^ SPSS^®^ Statistics GradPack 28).

### Ethical statements

In this study, the ethical principles that guide the research were followed, namely, the authorization of the Ethics Committees of the Faculty of Medicine of the University of Porto (reference 75/CEFMUP/2022) and the Regional Health Administration (reference 20220148). The respect and protection of all respondents were ensured by signing an informed, free and informed consent authorizing their participation in the study. Data confidentiality was guaranteed by delivering a document with information regarding the GDPR by Regulation 2016/679 of the European Parliament and the Council of the European Union on May 25, 2018.^21^ Personal data is kept for the period considered necessary for the investigation, respecting its conservation and the guarantees of secrecy and confidentiality required by the GDPR. The leading researcher and co-supervisor are professionals at the CPCT, where the study was carried out. However, they recognize their obligations of competence, honesty, reliability, objectivity, impartiality, and independence. They undertake to disseminate the results, according to these assumptions, with the rigor of science and for the benefit of the population, in the form of communications/posters in scientific forums and the form of publications of articles in magazines, independently, fulfilling their obligation as researchers to make research results publicly available.

## Results

Seventy-eight informal caregivers were selected according to the inclusion criteria, 15 of whom refused to participate in the study (8 refused to speak to the interviewer, and 7 did not respond because they found it difficult to talk about the death of their family member). Of the 63 caregivers who agreed to respond, 37 preferred to respond via email, and 26 chose to do so at home or in person. However, only 18 responses were received via email, and 26 were made in person.

The study surveyed 44 caregivers, of whom 93.2% were female. The majority were between 51 and 70 years old (65.9%) and married or in a civil partnership (54.5%). In terms of education, 47.8% had basic education, while 25% held a higher education degree (bachelor’s or master’s). Most caregivers were retired (43.2%) or actively working (40.9%). Nearly all (97.8%) lived in a house or apartment, and 88.6% identified as Catholic Christians. The primary kinship relationship was as a son or daughter (68.2%), followed by spouses (20.5%). Over half (52.3%) had a caregiving relationship lasting 41–60 years, and 68.2% had been providing care for more than a year. Table 1 contains a summary of the sociodemographic characteristics of the caregivers.

**Table 1.** Main sociodemographic characteristics of caregivers.

Sociodemographic characteristics	*N*	%
Age (years)		
19–30	1	2.3
41–50	4	9.1
51–60	15	34.1
61–70	14	31.8
71–80	8	18.2
81–90	2	4.5
Professional status		
Medical certificate	1	2.3
Active	18	40.9
Unemployed	6	13.6
Retired	19	43.2
Kinship		
Grandparents	1	2.3
Spouse	9	20.5
Other family member	3	6.8
Son/daughter	30	68.2
Without kinship	1	2.3
Length of relationship with the patient (years)		
<10	1	2.3
21–40	4	9.1
41–60	23	52.3
>60	16	36.4

*N*, number; %, percentage; <, inferior; >, superior.

Most informal caregivers provide care for a period exceeding 1 year (68.2%). The daily time spent was grouped into “less than 11 h” and “more than 11 h,” with 75% spending more than 11 h/day on care tasks, as shown in Table 2. These caregivers undertake tasks related to the basic and instrumental activities of daily living.

**Table 2.** Time and workload as a caregiver.

Time and workload	*N*	%
Time as a caregiver		
6–8 weeks	2	4.5
9–12 weeks	4	9.1
3–6 months	5	11.4
7–11 months	3	6.8
>1 year	30	68.2
Daily workload time as a caregiver (horas)		
<11	11	25
>11	33	75

*N*, number; %, percentage; <, inferior; >, superior.

When asked why they took care of the patients, 82.4% mentioned the relationship with the patient, and 32.4% referred to the feeling of duty. Monitoring the team and allowing the place of death to be at home was equally important in motivating people to take care of their relatives. Affection was mentioned by only two caregivers (5.9%).

Regarding challenges in providing care, emotional exhaustion was noticeably higher (84.1%), followed by physical exhaustion (75%). The study also allowed us to understand that managing the progression of the disease is a challenge for 63.3% of caregivers, and, finally, the financial strain was mentioned by 25.0%.

Regarding the main needs felt, the majority mentioned the need for help in providing care (59.1%), and a significant percentage felt the need for training (15.9%). However, it was possible to notice that the need for greater financial support was mentioned by 25.0%, coinciding with the same number of responses given to this challenge (financial exhaustion), as observed in Table 3.

**Table 3.** Challenges and needs identified by the caregiver.

Needs as a caregiver	*N*	%
Help with care	26	59.1
Training	7	15.9
24-h support from CPCT	1	2.3
Financial	11	25.0
None	6	13.6

CPCT, Community Palliative Care Team; *N*, number; %, percentage.

Due to the relevance that support networks demonstrate in their ability to complement the tasks inherent to care, both formal and informal support networks were studied. The results showed that the caregivers in the sample received more significant support from the informal support network (77.3%), of which the majority highlighted family members (81.8%), followed by support from friends (22.7%). It is also important to emphasize that 15.9% of respondents still need support from the informal support network.

Regarding the formal support network, the CPCT was highlighted by 93.2% of the sample, followed by the home support service (HSS) provided by Private Social Solidarity Institutions, with 11.3%.

We had no statistical significance regarding the relationship between the challenges experienced with the informal caregiver’s kinship (*p* = 0.345), financial strain (*p* = 0.262), physical exhaustion (*p* = 0.696), management of disease progression (*p* = 0.742), and exhaustion related to disease progression (*p* = 0.696).

Financial strain is relatively higher in caregivers who have been caregivers for longer (33.3% vs 7.1%), although the difference is not statistically significant (*p* = 0.076).

Regarding the place of care, 75% of caregivers provided care in the patient’s home, and 22.7% provided care in their own home.

Regarding the preference of place of death, 100% of patients had expressed, according to the caregiver, their preference for death at home. Regarding the place of death preferred by the informal caregiver, it was found that 93.2% preferred the patient’s or caregiver’s home. It was found that all patients had their real place of death at home, with 90.9% dying in the desired location.

The influence of the CPCT monitoring on the choice of place of death was also studied, with 72.7% of caregivers answering “yes” to the question “Influence of team monitoring on the choice of place of death.” As shown in Table 4, the dimensions of the team evaluation that were better evaluated were “The quality of care provided by the CPCT professionals” and “The knowledge/confidence felt in the CPCT professionals.” All aspects of the team’s evaluation received good evaluations as the respective averages are significantly higher than the average evaluation point of the scale (3 – Good; *p* < 0.001).

**Table 4.** Assessment of caregivers for CPCT.

Assessment of caregivers	Min	Max	*A*	SD
Technical knowledge of professionals	3	5	4.59	±0.69
Quality of care provided	3	5	4.64	±0.65
Knowledge/confidence felt	3	5	4.64	±0.65
Communication/established relationship	3	5	4.61	±0.65
Inclusion of the family in decisions	1	5	4.50	±0.90
Explanation of disease progression	2	5	4.43	±0.75
Availability/accessibility of professionals	3	5	4.52	±0.73
Team opening hours	2	5	3.80	±1.02
Telephone opening hours	2	5	3.93	±0.99

A, average; CPCT, Community Palliative Care Team; Max, maximum; Min, minimum; SD, standard deviation.

## Discussion

Regarding the profile of informal caregivers in the present study, they are between 45 and 75 years old with low educational qualifications, which aligns with the European profile outlined in the EuroCarers study, where the majority are also female.^22^ This information corroborates a Portuguese study carried out in 2021, which characterized informal caregivers similarly, where the percentage regarding gender was higher in females (81.3%), the age group highlighted was between 51 and 60 years, and with a level of education, mostly up to secondary education.^23^

It is important to stress that 4.5% of informal caregivers were between 81 and 90 years old, which may reflect the aging problem of the Portuguese society and changes in family size and composition, and the respective impact on home care.^2,23^

Regarding professional status, our results lead to a reflection on the impact of care on the personal and professional lives of caregivers since 75% (*n* = 33) report providing care for more than 11 h/day, and 68.2% (*n* = 30) have taken on the role of informal caregiver for more than a year. As this is a task where care is provided uninterruptedly, it may present difficulties in reconciling it with work activity. Our study aligns with previous studies on this subject.^22,23^

The informal caregiver status (ICS) promotes measures to reconcile the role of non-main informal caregiver with professional activity, namely: (1) you have the possibility of carrying out the activity on a teleworking basis if it is compatible with the activity you perform, schedule flexible, or part-time work; (2) annual leave of five working days to assist the person being cared for; (3) right to 15 days of justified absences in case of illness of the person being cared for. These measures are very recent, and their impact has not yet been assessed.^24^

The study mentioned above^23^ also refers to a characterization similar to the results obtained in this study regarding the degree of kinship and the reasons for assuming care. In the present study, most caregivers have had a relationship with the patient for more than 41 years, and this data confirms previous research that demonstrates the family’s responsibility for care.^16,25^

Our study is in line with a previous study that aimed to investigate the perceptions of informal caregivers about the daily care experience, referring to kinship, the relationship, respect for fulfilling the patient’s wishes, and the sense of duty as the main reasons for assuming care.^26^

Although the present study finds that the informal network is the main source of support received by informal caregivers, in accordance with data described in a previous study,^25^ with regard to the formal support network, the main support received by the interviewed caregivers was through the CPCT.

When asked about the challenges experienced as a caregiver, emotional exhaustion, physical exhaustion, management of disease progression, and financial strain are highlighted. Among the identified needs, assistance with care and financial support stand out. These data are in line with the results of other studies that indicate that informal caregivers face challenges that result in physical, emotional, social, and financial exhaustion.^27,28^

It should be noted that emotional exhaustion is the challenge most mentioned by informal caregivers (84.1%). These caregivers are exposed to various risks, namely due to the overload of caregiving tasks, continuous care, and social deprivation. The emotional exhaustion of informal caregivers has been studied by several authors^27,29,30^ who demonstrate a need to define effective support strategies that respond to their needs.

It cannot be forgotten that caregivers are mostly elderly spouses who also have multiple health problems and are often frail elderly. Everyone, patients, families, and caregivers, suffer in a multidimensional way. The emotional suffering that was clearly highlighted in this study will be influenced by physical, psychological, social, cultural, work-related, and spiritual suffering. What our study also shows is that these caregivers feel very helpless, recognizing in the team their greatest source of comfort, but they should have more economic and social support from the State, as they ensure the efficiency of the health system and quality of care. On the other hand, these problems are not new, given that we are dealing with patients with progressive chronic diseases. Therefore, psychological support should begin earlier, and the health system should provide this support before it is necessary to integrate patients into specialized PC teams.

In this context, there is a response from the Portuguese National Integrated Continued Care Network to provide periods of relief or adequate rest for caregivers, temporarily freeing them from the tasks inherent to providing care.^31^ However, this measure presents limitations in the referral criteria and admission time. The Palliative Care Units (PCUs) also have a caregiver rest measure for patients monitored in PC. However, in addition to the constraint of waiting to enter due to the insufficient number of beds, there is the constraint of the geographic area of the PCUs.

It was also noted that emotional exhaustion and kinship with the patient did not present a statistically significant relationship. However, the emotional burden of patients in palliative situations and their families takes on specific characteristics, requiring special attention to this fact.

In this study, 25% of respondents mentioned financial difficulties as being a challenge and a necessity, a difficulty that can be caused by their employment situation and the increased expenses that the illness entails. Likewise, the EuroCarers study^22^ states that, in 59.8% of its participants, the increase in monthly costs was one of the foremost leading financial repercussions felt by caregivers when providing care. The results of the present study show that 43.2% of caregivers were retired and 13.6% were unemployed, which may also represent an additional problem in terms of the family budget. As caregivers are not paid for the work they provide, there is a very high economic impact, both due to the hours spent on tasks and the expenses they incur to provide care.^32^

By Portuguese Law No. 100/2019, of September 6th, the informal caregiver must live in the same house as the person being cared for, cannot carry out paid professional activity, and provide care permanently.^33^ Being an advance in increasing the rights, recognition, and visibility of informal caregivers, it presents several limitations, particularly with regard to the financial strain that care entails. It establishes that the caregiver does not carry out a professional activity or another type of activity incompatible with the provision of permanent care to the person being cared for, as well as not receiving pensions or unemployment benefits. When financial support is identified by 25% of respondents as a need associated with care, this situation leads to reflection on the feasibility of the ICS in terms of responding to the practical needs felt by caregivers.

Therefore, it is highlighted that the work carried out by informal caregivers should contribute to the planning of social policies since it is estimated that the value of the work carried out by them in Portugal corresponds to 2% of the Gross Domestic Product.^34^ The importance of IC still needs to be addressed, whether in the provision of direct or other care, but also in the implications for the country’s economy and development.

Nearly 70% of caregivers identified training to perform the difficult task of care as a need in a study that characterized the profile of caregivers at a national level.^23^ In the present study, 15.1% of caregivers also identified it as a need. Therefore, we should have an education plan focused on the knowledge needs of a caregiver.

In the present study, 75% of respondents present physical exhaustion as their main challenge, and 59.1% identify help with care as their main need. These results point to the insufficiency of social responses and the fact that they are focused on care rather than the caregiver. By way of example, the HSS recommended by Portuguese legislation is restricted to the provision of hygiene and personal comfort care, provision and support with meals and laundry treatment, and this care is provided in a detailed manner and in a time-limited exclusively for the task.^35^

There are challenges and needs listed by caregivers regarding support networks, whether formal or informal. The informal caregivers in this study report receiving support from formal and informal support networks (45.5% and 77.3%, respectively). Regarding the informal network, 81.8% of respondents received support from family, 22.7% from friends, and 9.1% from neighbors. Regarding care from the formal support network, 93.2% refer to CPCT, and 11.3% refer to the HSS. These results suggest that CPCT has a positive impact on informal caregivers, which is in line with other international studies.^10,16^

The results also highlight the optimistic assessment that informal caregivers made of the CPCT in several dimensions. It can be seen that all aspects of the CPCT evaluation received good evaluations, as the respective averages are significantly higher than the midpoint of the scale (3 – Good). Of all dimensions that stand out in the study are “quality of care provided by the CPCT” and “knowledge felt by CPCT professionals,” with an average rating of 4.64. The quality of the relationship between healthcare professionals, patients, and family members was an important factor in the selection of end-of-life care. The literature states that clear transmission of the clinical situation facilitates decision-making about the place of care and place of death.^7,16,36^

On the other hand, the worst-rated dimensions were telephone service hours and opening hours, with a rating of 3.93 and 3.80, respectively. This result should be the subject of reflection by team members and the management structures, as opening hours are from 8:00 am to 7:00 pm, Monday to Friday. Nights and weekends are periods when the patient and the informal caregiver do not have the support of the team, which could increase the need for hospital emergencies. International literature refers to the need to implement home PC teams with 24-h opening hours, 7 days a week, to avoid readmissions, which, in turn, are predictors of hospital death.^11^

Like this, the operation of community PC teams 24 h and 7 days a week is necessary to increase deaths at home if that is the desire of the patient and their family.^9,11,16,37^ However, in a recent study published by this team,^19^ it seems that this is a team that safeguards excellent training for caregivers, particularly the management of uncontrolled symptoms with *pro re nata* medication, with patients being monitored showing a very low emergency recurrence rate (only 37 recurrences in 450 patients followed in the year 2023). Despite being a point of possible improvement to increase health value in relation to the feeling of security on the part of caregivers, the truth is that the team observes good behavior after training in managing unexpected situations.

In this sense, and after this study, a document was prepared advising the hiring of more medical and nursing professionals so that it is possible to carry out a pilot evaluation with the implementation of telemedicine in those periods without the possibility of direct assistance support and, if there are results in this sense, we can evolve to an assistance support system available 24/7.

Scientific evidence shows that patients who express their preferences for end-of-life care are more likely to have these preferences respected.^38^ As already mentioned, it appears that most people want to die at home.^7^ The respondents of this study confirmed this fact since all patients expressed their desire to die at home, according to the caregivers. As a limitation of the study, these preferences were documented indirectly and retrospectively. Therefore, the reported preferences may be subject to recall bias. Home is considered a place that provides security and a greater sense of control, and where family members generally provide care.^39,40^ When observing the results of the questionnaires of the informal caregivers surveyed, it appears that 93.2% showed a preference for home as the place of death of the patient. It was also found that the place of death desired by caregivers occurred in 100% of cases (at home), and the place of preference of patients occurred in 90.9% of cases (at home). These results are very significant, given that in the aforementioned European study, only a third of patients died at home.^4^ Given the importance of follow-up by PC, particularly the home team, and the fact that only 14% of European patients receive a response from PC teams,^6^ it is clear that the study population is privileged and would be expected to have this potential for death at home.

The congruence between the preference for place of death and the place of occurrence is an important indicator of the quality of healthcare provided to patients^40^ at the end of life.^10,11^ The results of this study showed that the preferences of the patient and the informal caregiver regarding the place of death were strongly coincident. Furthermore, for the patient’s preference for home care to be respected, the family’s decision for the last place of care seems to be fundamental.^7^ It is noteworthy that 72.7% of respondents report that CPCT monitoring had a significant influence on the choice of place of death. It is also worth highlighting the reference in the literature to the quality of the relationship between both (caregiver and patient), evidenced by solid emotional relationships, the availability of some member of the family to provide support to the patient, internal resources from the family itself, and the existence of external resources from the community.^7,17^

Although in our study, no patient translated their wishes regarding the health care they wanted to receive, the literature mentions that advance directives are also a predictor of death at home.^13^

The results of this study should not be transferred to other realities, even taking into account the existence of home PC teams, as the characteristics of the population, local geographic characteristics, the characteristics of each professional on the team, and its 5 years of activity, with a large investment in the training of family health teams and intervention in the community, make this project difficult to compare with the results regarding satisfaction with the work of the team’s professionals.

## Limitations

Being a retrospective study, which depends on the opinion of informal caregivers, they may be prone to memory bias. Therefore, the data used may not be accurate according to past events.

It is also considered that the sample size was a limitation, as its small size resulted in some limitations in statistical treatment, namely, the finding of significant correlations and the probability of essential generalizations to respond to some objectives, such as identifying factors that influenced the chosen place of death. As already stated, the patient’s preferred place of death may be subjected to recall bias. A larger sample may lead to significant differences in some evaluated parameters. It has already been mentioned that PC research presents methodological challenges due to different factors, such as vulnerability and the existence of delicate topics that take more work to approach. This could have been the reason for the lack of some responses.

Another relevant limitation of this study is the fact that it is unicentric, using patients monitored by the same community PC team, with no control or comparison with other teams.

## Conclusion

This is one of the first studies carried out that we know of to investigate the challenges of informal caregivers for patients in PC, comparing them with the place of death, but also with other fundamental dimensions for the necessary balance when caring, at home, namely the support provided by the informal and formal network.

The impact of monitoring the CPCT is highlighted, as evidenced by the assessment carried out by informal caregivers. It is also the only study that evaluates the caregiver’s perspective in the *post-mortem* that we are aware of and that addresses patients who died, regardless of the pathology, and without selection bias.

We conclude that these findings underscore the importance of home-based PC services in respecting patient and caregiver preferences for death at home while also highlighting the need for enhanced support systems to address the challenges faced by informal caregivers.

## Supplemental Material

sj-docx-1-pcr-10.1177_26323524251336764 – Supplemental material for The influence of informal caregivers on the place of death: A study within a home palliative care team Supplemental material, sj-docx-1-pcr-10.1177_26323524251336764 for The influence of informal caregivers on the place of death: A study within a home palliative care team by Cristina Pereira, Hugo Ribeiro, Júlia Magalhães, João Rocha-Neves, Marília Dourado and José Paulo Andrade in Palliative Care and Social Practice

## Figures and Tables

**Table:** 

	Bad	Reasonable	Good	Very good	Excellent
27. The technical knowledge of CPCT professionals	1	2	3	4	5
28. The quality of care provided by CPCT professionals	1	2	3	4	5
29. The trust felt in CPCT professionals	1	2	3	4	5
30. The communication/relationship established with CPCT professionals	1	2	3	4	5
31. The way the family was included in decisions	1	2	3	4	5
32. The way in which the progression of the disease was explained	1	2	3	4	5
33. The availability/accessibility of CPCT professionals	1	2	3	4	5
34. CPCT opening hours	1	2	3	4	5
35. CPCT telephone opening hours	1	2	3	4	5

## Annex – English translation of the questionnaire that was used

### The perspective of the informal caregiver and their influence at the place of death

This survey is being carried out with the aim of identifying factors that influence the place of death and maintenance of the place of death in patients monitored by the CPCT that accompanied their family members.

Your participation in this study will last approximately 30 min.

Please note that all information collected will be treated confidentially and with the sole purpose of carrying out this investigation.

The response to this survey will be in physical format.

You must complete the Informed Consent document. Data confidentiality is guaranteed.

1. Gender
□ Female
□ Male
□ Nonbinary
□ I prefer not to answer
2. Marital status
□ Single
□ Married
□ Separate
□ Divorced
□ Widower
□ Other:___________
3. What is the highest level of education you have completed?
□ None
□ Basic education first cycle
□ Basic education second cycle
□ Basic education third cycle
□ Secondary education
□ Non-higher technological specialization courses
□ Higher professional technical course
□ Bachelor’s degree
□ Graduation
□ Master’s degree
□ Doctorate
4. Age
□ 19–30 years
□ 31–40 years
□ 41–50 years
□ 51–60 years
□ 61–70 years
□ 71–80 years
□ 81–90 years
□ More than 90 years
5. Professional situation
□ Active
□ Unemployed
□ Retired
□ Other: ____________
6. Housing situation
□ House
□ Apartment
□ Other: ______________
7. Do you belong to a religion or religious/spiritualist group?
□ Catholic Christian
□ Protestant Christian
□ Orthodox Christian
□ Jehovah’s Witness
□ Jewish
□ Muslim
□ No religion (includes agnostic and atheist)
□ Other: __________
8. Degree of kinship with the patient:
□ Spouse
□ Father or Mother
□ Grandfather or Grandmother
□ Other family member: ________________
□ Without kinship
9. Length of relationship between the caregiver and the patient
□ <10 years
□ 11–20 years
□ 21–40 years
□ 41–60 years
□ >60 years
10. Place of care
□ Patient’s home
□ Caregiver’s home
□ Other: ____________
11. Preference for the patient’s place of death
□ Home
□ Palliative care unit
□ Hospital
□ Nursing home
□ Do not know
□ Other: _____________
12. Caregiver’s preference for place of death
□ Home
□ Palliative care unit
□ Hospital
□ Nursing home
□ Do not know
□ Other: ____________
13. The patient died in the desired location?
□ Yes
□ No
14. Factors that influenced the choice of place of death
□ Relationship with the patient
□ Affection
□ Feeling of duty
□ Monitoring by CPCT
□ Other: ________
15. The caregiver’s preference for place of death and the patient’s preference coincided?
□ Yes
□ No
16. If you answered no to the previous question (question 15), why?
□ Clinical criteria
□ Financial condition
□ Caregiver’s burden
□ Lack of training
□ Other: ____________
17. The patient had a living will or advance directives?
□ Yes
□ No
18. What motivated you to become a caregiver?
□ Relationship with the patient
□ Allow the place of death to occur at home
□ Monitoring by CPCT
□ Duty
□ Affection
□ Other: ____________
19. Time as a caregiver:
□ Less than 2 weeks
□ 2–5 weeks
□ 6–8 weeks
□ 9–12 weeks
□ 3–6 months
□ 7–11 months
□ More than 12 months
20. Time spent on tasks:
□ Less than 1 h/day
□ 1–2 h
□ 3–4 h
□ 5–6 h
□ 7–8 h
□ 9–10 h
□ More than 11 h/day
21. Who gave you most support?
□ Family
□ Professional help
□ Neighbors
□ Others
22. Support from the informal network (select all that apply)
□ Family members
□ Friends
□ Neighbors
□ Others: ______________
23. Formal network support (select all that apply)
□ Home support service
□ Associations
□ Church
□ CPCT
□ Local commerce
□ Other: _____________
24. What were the main challenges associated with the role of caregiver (select all that apply)
□ Financial
□ Physical burden
□ Emotional burden
□ Management of disease progression
□ Other: ________________
25. What help would have been important to you?
□ Financial support
□ Help with care
□ Training
□ Other: __________
26. Influence of team monitoring on the choice of place of death
□ Yes
□ No

For each of the following questions, select the answer that best fits your opinion.

*How do you classify*:

36. Did you feel heard by CPCT professionals?
□ Never
□ Rarely
□ Occasionally
□ Often
□ Very often
37. If you needed to contact a team professional at night, would you have that option?
□ Yes
□ No
38. If you needed to contact a team professional during the weekend, would you have that option?
□ Yes
□ No
